# Isolated Cardiac Sarcoidosis Masquerading as Peripheral Vertigo: A Case Report

**DOI:** 10.7759/cureus.83710

**Published:** 2025-05-08

**Authors:** Sai Amruth Boppe, Shashikala T Surkunda, Weena Stanley, Arjun Ballal

**Affiliations:** 1 Department of Internal Medicine, Kasturba Medical College, Manipal, Manipal, IND; 2 Department of Medicine, Kasturba Medical College, Manipal, Manipal, IND

**Keywords:** av block, cardiac arrhythmias, cardiac fdg-pet, cardiac mri, giddiness, granulomatous inflammation, icd, isolated cardiac sarcoidosis, non sustained ventricular tachycardia, vertigo

## Abstract

Cardiac sarcoidosis is a rare cause of cardiomyopathy and is especially difficult to diagnose when there is no pulmonary involvement in the patient. Here, we present a case of a 70-year-old male nil premorbid patient who presented with complaints of giddiness and fatigue. The episodes of giddiness were characterized as a feeling of intense sensation of imbalance as if he was falling forward, which was initially diagnosed as peripheral vertigo after thorough evaluation. The cardiac causes were initially ruled out as he had no symptoms of chest pain, dyspnea, or palpitations; his cardiac physical examination was completely normal, and echocardiography showed normal biventricular systolic function with no wall motion abnormalities. With time, new symptoms appeared, such as chest pain and fatigue, which made the patient present to the Emergency Medicine Department (EMD), where the cardiac echocardiography revealed a possible scar, and a cardiac MRI, which was done post-echo, showed continuous epicardial late gadolinium enhancement along the lateral, inferior, and anterior walls of the mid and basal cavities of the left ventricle. An FDG PET-CT (Fluorodeoxyglucose Positron Emission Tomography-Computed Tomography) scan showed diffuse hypermetabolism of the left ventricle, which narrowed the diagnosis to cardiac sarcoidosis. After the implantable cardioverter defibrillator (ICD) insertion alongside corticosteroids and antiarrhythmics, the patient had a dramatic improvement in symptoms. This case highlights the importance of timely and repeated cardiac evaluations and the use of advanced imaging techniques in patients with unexplained presyncope associated with conduction abnormalities.

## Introduction

Sarcoidosis is a granulomatous disorder that causes non-caseating granulomas in any organ in the body. It starts due to a trigger such as an infection wherein there is an uncontrolled inflammatory response with the activation of macrophages and CD4+ T cells, leading to granuloma formation. These granulomas can commonly affect the lungs, skin, and eyes. Other organs that can be affected include the heart, liver, and brain. Approximately 25% of patients have isolated cardiac sarcoidosis without systemic involvement [1].

Cardiac sarcoidosis (CS) can cause heart failure, sudden cardiac death, arrhythmias (such as atrial fibrillation or ventricular arrhythmias), myocardial infarction, or atrioventricular block (AV Block) [1]. Conduction system abnormalities occur due to granuloma formation in the interventricular septum, disrupting the conduction pathway. The most common presentations are conduction system disorders and heart failure [2]. The signs and symptoms include palpitations, presyncope, and syncope, but they can also cause more subtle symptoms, such as fatigue and exertional dyspnea. CS is commonly diagnosed late due to these nonspecific symptoms.

In this case, the patient presented with the symptoms of giddiness and fatigue. His giddiness was a complaint of episodes of imbalance, in which he felt like he was falling forward. After ruling out cardiac involvement through patient history, clinical examination, and diagnostic tests such as echocardiography, which all turned out to be expected, he was suspected of having peripheral vertigo. First, a neurology consult was sought to evaluate vertigo, where they ruled out the central causes with the help of an MRI of the brain. Then, an ears, nose, and throat (ENT) consult was called for in view of suspected peripheral vertigo, where they advised conservative management with labyrinthine sedatives. As his giddiness improved with conservative management, the case was dismissed as a minor illness. Months later, he presented to the emergency department with new complaints of chest pain and fatigue, in addition to his previous symptom of giddiness. As the patient developed new symptoms, a thorough evaluation was done again. Although his labs were normal, his electrocardiogram (ECG) showed Q wave, R wave, and S wave (QRS) notching in leads 2,3, and augmented voltage foot (AVF). Following this, an echocardiography was done, which revealed hyperechogenicity of the mid-distal inferior wall. Both these findings could be attributed to a scar. This significant finding led to further investigations, such as a cardiac MRI and FDG PET-CT. The findings on these tests, when combined with his clinical symptoms, lead to the possibility that he might have cardiac sarcoidosis and that his initial complaints of episodic imbalance were pre-syncopal and may have been caused by ventricular arrhythmias.

Cardiac sarcoidosis (CS) is considered a diagnosis of exclusion as there is no single diagnostic laboratory or radiologic test to confirm the cause. The most accurate way to diagnose CS is by combining multiple factors to narrow the diagnosis. These include clinical features such as ventricular dysfunction, ventricular arrhythmias, or complete heart block, using imaging modalities like FDG-PET, cardiac MRI, and a myocardial biopsy showing noncaseating granulomas if possible. If untreated, CS can lead to dilated cardiomyopathy or sudden cardiac death due to life-threatening arrhythmias [1]. This case highlights the importance of including CS in the differential of unexplained ventricular tachycardia with presyncope.

## Case presentation

A 70-year-old male patient presented to the outpatient department with complaints of giddiness, acute in onset, with 1-2 episodes occurring per day; there was no postural variation. He first noticed it while taking a shower and described it as a sensation of imbalance and unsteadiness, which made him feel like he was falling forward. The episodes were resolved sitting down. He had no history of chest pain, palpitations, loss of consciousness, swaying while walking, atrial fullness, reduced hearing, tinnitus, earache or ear discharge, abnormal sweating, bowel or bladder disturbances, and premature ejaculation. On general examination, he had mild pallor and a regular pulse but no postural drop in blood pressure. On systemic examination of the chest, there were no murmurs or added sounds, and neurological examination revealed no focal neurological deficit. Laboratory investigations showed mild iron deficiency anemia, normal serum plasma sugars, electrolytes (sodium, potassium, calcium, magnesium), cortisol, vitamin B12 levels, cardiac markers (troponin T), and thyroid profile. Cardiovascular evaluation (electrocardiogram (ECG), 2D echocardiography, and bilateral vertebral carotid doppler) revealed no significant abnormal findings. Ambulatory cardiac monitoring detected a sinus rhythm with first-degree Atrioventricular (AV) block and ventricular and supraventricular ectopic beats. Audiological evaluation by the ENT department found reduced hearing in his right ear. As thorough examination and investigations could help rule out cardiac etiologies, he was initially diagnosed with peripheral vertigo and iron deficiency anemia. His symptoms improved with labyrinthine sedatives, and therefore, he was discharged on tapering doses of the same with hematinics and was advised to consult ENT at follow-up. Retrospectively, his ectopic beats, which were not given much thought and were initially ruled out as harmless, could have been due to the myocardium that was affected by granulomas.

**Figure 1:**
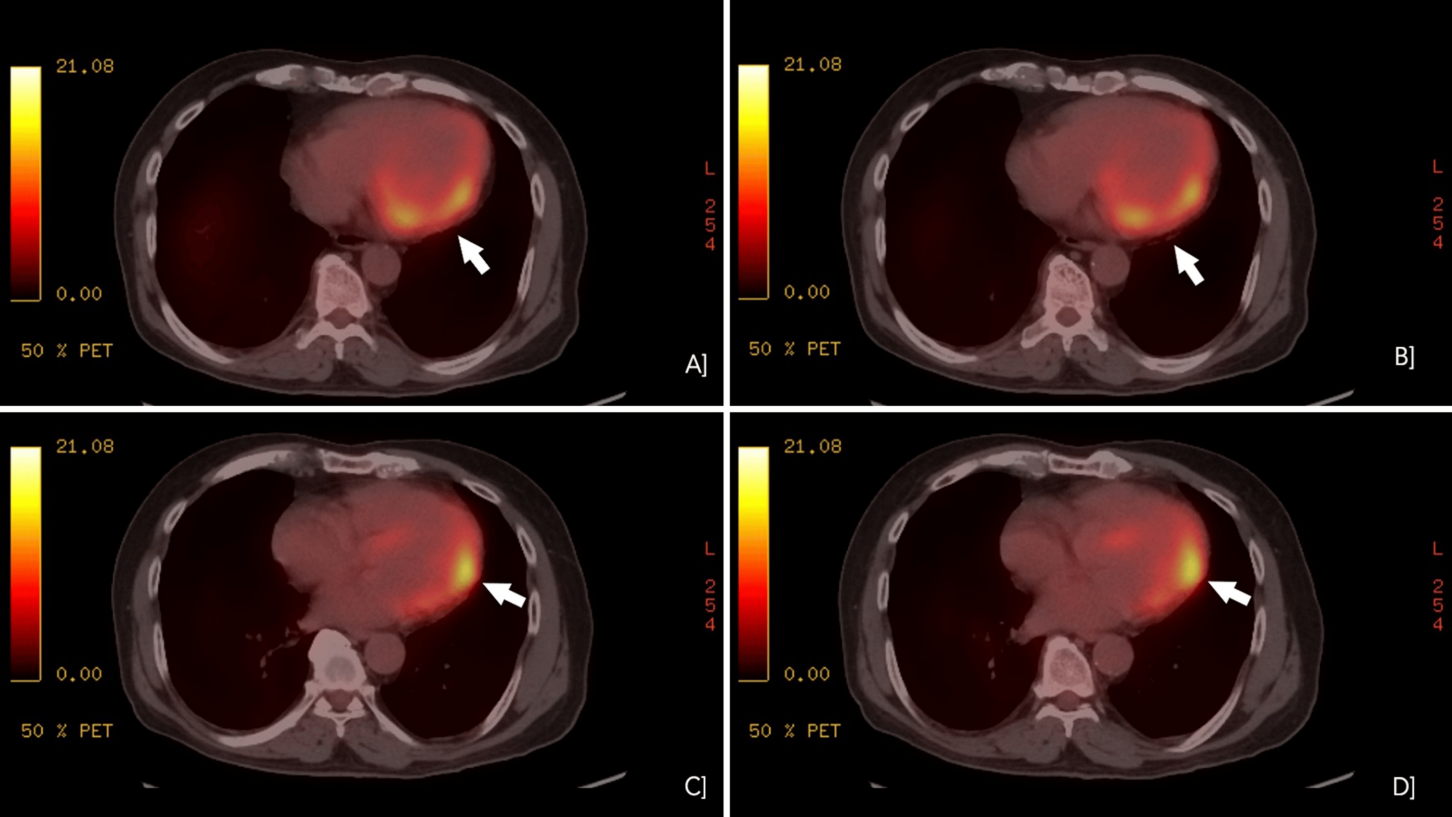
Axial view of different slices of FDG PET-CT showing diffuse hypermetabolism of the anterolateral wall of left ventricle FDG PET-CT: Fluorodeoxyglucose positron emission tomography-computed tomography

**Figure 2:**
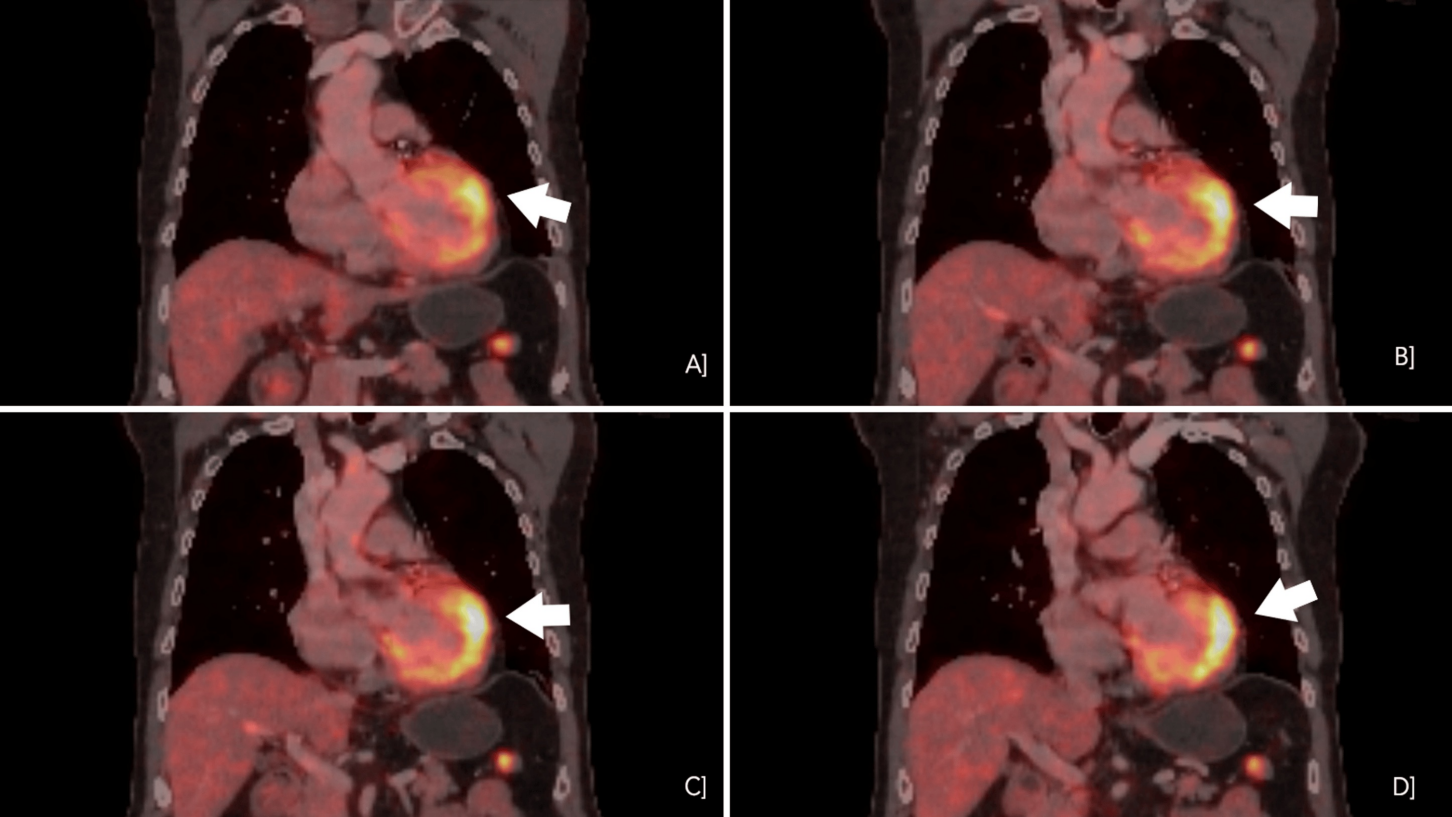
Coronal view of different slices of FDG PET-CT showing diffuse hypermetabolism of the anterolateral wall of left ventricle FDG PET-CT: Fluorodeoxyglucose positron emission tomography-computed tomography

His labyrinthine sedatives helped alleviate symptoms until six months later, when he arrived at the emergency medicine department (EMD) with giddiness, chest pain, and fatigue. This progression of symptoms made the treating physician doubt their initial diagnosis. His preliminary investigations revealed normal hemoglobin, serum electrolytes, and cardiac markers. His serum angiotensin-converting enzyme (ACE) levels were within the normal range at 12 U/L, though elevated levels can be seen in sarcoidosis. Electrocardiogram (ECG) showed notching of QRS in leads 2, 3, AVF, and echocardiography (ECHO) revealed hyperechogenicity of mid-distal inferior wall (suspected scar), with an ejection fraction of 55%. Repeat ambulatory cardiac monitoring revealed recurrent episodes of non-sustained ventricular tachycardia. A treadmill stress test induced rapid, non-sustained ventricular tachycardia. Coronary angiography showed single-vessel disease in the left anterior descending artery (50% occlusion), which did not require a stent. Unconvinced that coronary artery disease was the cause of his symptoms, cardiologists recommended a cardiac MRI, which revealed "continuous epicardial late gadolinium enhancement along the lateral, inferior, and anterior walls of the mid and basal cavities of the left ventricle, without involvement of the subendocardial layer." This finding indicated the presence of myocardial fibrosis in these regions, but since the subendocardial layer was unaffected, it was not due to ischemia. Confirming the suspicion that coronary artery disease was not the underlying cause, an FDG PET-CT was performed, which showed "focal on mild diffuse hypermetabolism involving the anterolateral wall of the left ventricle (Figures 1A-1D) (Figure 2A-2D), with no other visceral involvement". The images included in this case report highlight focal areas of increased fluorodeoxyglucose (FDG) uptake without any significant perfusion defect. These findings suggest an inflammatory etiology rather than myocardial infarction, as active infarction-related inflammation would typically demonstrate reduced perfusion. Focal hypermetabolism also helps us rule out myocarditis, which commonly presents as a diffuse hypermetabolism without any focal uptake on FDG PET-CT. The hallmark findings included non-sustained ventricular tachycardia, epicardial late gadolinium enhancement on cardiac MRI, and focal to diffuse hypermetabolism on FDG PET-CT, consistent with cardiac sarcoidosis. With these findings, he was diagnosed with isolated cardiac sarcoidosis as the clinical and diagnostic findings correlated, and he fit the Japanese Circulation Society diagnostic criteria for it. His management included corticosteroids, bisoprolol and amiodarone. The FDG PET-CT findings of hypermetabolism justified the initiation of high-dose corticosteroids to control active inflammation. They reduce inflammation and granuloma formation in the myocardium and are the first line of management of CS. He was started on Prednisolone 30mg/day and slowly tapered to 7.5mg/day in six months. Bisoprolol helps regulate the heart rate, and amiodarone helps regulate the rhythm, decreasing the possibility of atrial fibrillation. Since this patient had ectopic beats in the past that could, in the future, progress to atrial fibrillation, this management was necessary. Finally, an implantable cardioverter-defibrillator (ICD) implantation was done, as it was indicated in this patient who had cardiac sarcoidosis with unexplained syncope and recurrent episodes of non-sustained ventricular tachycardia. The patient's symptoms significantly improved with the above measures, and he was discharged on the above medications with a tapering dose of steroids. At the follow-up, the patient was symptom-free, and a repeat echocardiogram showed no further deterioration in echocardiographic parameters. With the initial worry of further progression being halted and patient symptoms relieved, he was put on a monthly follow-up and told to come back immediately in case of any new symptoms.

## Discussion

CS is commonly underdiagnosed due to its vague presentation in its early stages. These include symptoms such as chest pain, palpitations, fatigue, breathlessness, or episodes of loss of consciousness that can overlap with cardiac conditions such as myocardial infarction, atrial fibrillation, valvular disease, and systemic conditions such as Meniere's disease or vertigo. This overlap was one of the main reasons the physicians found it challenging to diagnose the patient effectively during the first visit. The clinical manifestations depend on the location and size of the granuloma involving the myocardium [3]. Serum angiotensin-converting enzyme (ACE) levels are elevated in 60-80% of sarcoidosis cases. However, this is a non-specific finding, as other diseases can cause elevated ACE levels. The diagnostic criterion for cardiac sarcoidosis is often based on guidelines from the Heart Rhythm Society (2014) or the Japanese Circulation Society (JCS) (2016). These criteria have a minimum required fulfillment to establish a clinical diagnosis of CS. The significant difference among them is that the Heart Rhythm Society (HRS) criterion requires some histological proof of sarcoidosis that can be a cardiac or extracardiac finding, whereas the JCS guidelines do not [4,5]. As endomyocardial biopsy has a low sensitivity (less than 20%) due to the focal nature of the disease [6] and carries a risk of serious post-procedural complications, and since the clinical criteria for a diagnosis of cardiac sarcoidosis had already been fulfilled according to the Japanese Circulation Society (JCS) guidelines, the decision was made to forgo the biopsy. JCS and HRS (2014) criteria utilize efficient diagnostic imaging tools such as cardiac MRI or FDG PET-CT. These imaging modalities highlight areas of scarring/fibrosis or increased uptake of radiotracers, respectively, that can be attributed to inflammation. In our case, the images in the case report showed focal areas of increased FDG uptake without corresponding perfusion defects. This pattern points toward an inflammatory process rather than a myocardial infarction, which usually shows reduced perfusion in affected areas. The presence of focal hypermetabolism also makes myocarditis less likely, as that typically presents with a more diffuse uptake pattern on FDG PET-CT. Our case diagnosis is based on the JCS (2016) guidelines, as the patient's findings met two of the five major criteria of the JCS (2016) guidelines: Cardiac MRI with late gadolinium enhancement and FDG-PET showing high tracer uptake in the myocardium.

The initial clinical signs of cardiac sarcoidosis often include conduction abnormalities, such as AV block or ventricular tachycardia, both of which were present in our patient [7,8]. His giddiness can be attributed to multiple explanations, such as ventricular tachycardia or cardiac block, which could have decreased his cardiac output. CS needs to be detected early as immunosuppressive therapy does not improve the left ventricular function of patients with severely reduced left ventricular ejection fraction (LVEF) (<30%) due to a higher scar burden [9]. Congestive heart failure accounts for 25-75% of all cardiac-related deaths in patients with CS [10]. However, sometimes, before a patient progresses to this stage, he can have a fatal arrhythmia. Therefore, a multi-faceted approach that includes correlating clinical signs and symptoms and results from practical diagnostic tests such as ECG, echocardiography (ECHO), and FDG-PET scan should be employed to efficiently diagnose a patient early.

Management of patients with CS has remained consistent throughout the years. Once diagnosed, management consists of two main components: first, halting the progression of sarcoidosis with corticosteroids or other immunosuppressants, such as anti-tumor necrosis factor (TNF) agents and methotrexate. Immunosuppressants work by decreasing cytokine production, which helps in halting granuloma formation and inflammation. In return, this helps decrease susceptibility to high-grade AV block and causes recovery of left ventricular function. The second component focuses on controlling conduction abnormalities with antiarrhythmic medications [11]. Lastly, an implantable cardioverter-defibrillator (ICD) was indicated in our patient due to his diagnosis of cardiac sarcoidosis and the presence of non-sustained ventricular tachycardia, which constitutes a Class IIa indication for ICD implantation. This helps prevent sudden cardiac death. One of the significant drawbacks regarding the management of CS is the presence of only a small amount of data regarding the topic, because of which there is a large variability in the dosing and duration of drugs due to varied opinions. Ideally, as the duration of treatment is long, a preplanned transition of corticosteroids to steroid-sparing agents must be done to prevent cardiac toxicity. Follow-up with regular FDG-PET scans, cardiac magnetic resonance imaging (CMR), and echocardiography to monitor immunosuppression progression is important, too.

The typical patient affected by CS is a known case of sarcoidosis that, on routine screening or due to clinical symptoms, was found to have cardiac involvement. Recent studies have mainly focused on the different conduction abnormalities related to the condition in patients with isolated CS, with many patients often presenting multiple symptoms such as palpitations, sweating, shortness of breath, and chest discomfort. In contrast, our patient experienced only two symptoms: giddiness and fatigue for six months, which improved with labyrinthine sedatives, after which his other symptom was vague chest pain. Hence, this presentation can be considered as "atypical". Therefore, this case report emphasizes the importance of correlating subtle yet significant findings with abnormal diagnostic results to ensure that treatment targets the underlying disease rather than merely addressing the symptoms.

## Conclusions

This case report highlights the importance of considering cardiac sarcoidosis as a potential differential diagnosis in patients presenting with presyncope of unknown origin, especially in the geriatric population. It reflects the challenges of timely and accurate diagnosis in atypical presentations. At geriatric age (65+ years), fatigue-like symptoms are commonly chalked up to aging. Other symptoms, such as chest pain, shortness of breath, or foot swelling, are assumed to stem from common cardiac conditions like chronic heart failure and are promptly treated symptomatically. Very rarely is a deep dive done on the reason for the person's cardiac symptoms; hence, reversible causes such as early CS can easily be missed. According to the Heart Rhythm Society (2014), screening patients with unexplained third-degree AV block or sustained monomorphic VT due to unknown etiology has proved helpful [12]. In our case, the diagnostic process was challenging due to the absence of pulmonary involvement. The patient's progressive symptoms, ranging from giddiness to chest pain and fatigue, pointed toward a cardiac etiology. However, it was only through diagnostic imaging that the actual underlying condition was revealed. With advancements in diagnostic imaging techniques such as CMR or FDG PET-CT, easier and faster diagnoses can be made. Cardiac MRI can detect early wall edema and late myocardial scarring through late gadolinium enhancement, while FDG PET-CT highlights areas of active inflammation. High vigilance and repeated cardiac evaluation when in doubt can be lifesaving, as untreated active cardiac sarcoidosis can lead to progressive granulomatous infiltration of myocardial tissue, resulting in life-threatening arrhythmias, sarcoid myocarditis, or congestive heart failure.
